# Sensitive detection of viable circulating tumor cells using a novel conditionally telomerase-selective replicating adenovirus in non-small cell lung cancer patients

**DOI:** 10.18632/oncotarget.16818

**Published:** 2017-04-04

**Authors:** Shinsaku Togo, Nobuyoshi Katagiri, Yukiko Namba, Miniwan Tulafu, Kumi Nagahama, Kotarou Kadoya, Kazuya Takamochi, Siaki Oh, Kenji Suzuki, Fuminori Sakurai, Hiroyuki Mizuguchi, Yasuo Urata, Kazuhisa Takahashi

**Affiliations:** ^1^ Department of Respiratory Medicine, Juntendo University School of Medicine and Graduate School of Medicine, Tokyo, Japan; ^2^ Research Institute for Diseases of Old Ages, Juntendo University Graduate School of Medicine, Tokyo, Japan; ^3^ Oncolys BioPharma, Inc., Tokyo, Japan; ^4^ Department of General Thoracic Surgery, Juntendo University School of Medicine, Tokyo, Japan; ^5^ Laboratory of Biochemistry and Molecular Biology, Graduate School of Pharmaceutical Sciences, Osaka University, Osaka, Japan

**Keywords:** circulating tumor cells, EMT, telomescan, non-small cell lung cancer, EpCAM

## Abstract

Circulating tumor cells (CTCs) have a crucial role in the clinical outcome of cancer patients. Detection of non-small cell lung cancer (NSCLC) using an antibody against epithelial cell adhesion molecule (EpCAM) in captured CTCs has low sensitivity; the loss of epithelial markers leads to underestimation of CTCs with mesenchymal phenotype. We propose a new approach for detection of viable CTCs, including those with epithelial-mesenchymal transition status (EMT-CTCs), using the new telomerase-specific replication-selective adenovirus (OBP-1101), TelomeScan F35. Peripheral venous blood samples and clinicopathological data were collected from 123 NSCLC patients. The sensitivity of CTC detection was 69.1%, and for patients with stage I, II, III and IV, it was 59.6%, 40.0%, 85.7%, and 75.0%, respectively. Among the EMT-CTC samples, 46% were vimentin positive and 39.0% of non-EMT-CTC samples were EpCAM positive. Patients testing positive for EMT-CTCs at baseline had poor response to chemotherapy (*P* = 0.025) and decreased progression-free survival (EMT-CTC positive vs. negative: 193 ± 47 days vs. 388 ± 47. days, *P* = 0.040) in comparison to those testing negative. TelomeScan F35 is a highly sensitive CTC detection system and will be a useful screening tool for early diagnosis of NSCLC patients. Mesenchymal-phenotype CTCs are crucial indicators of chemotherapeutic efficacy in NSCLC patients.

## INTRODUCTION

Metastasis-related events contribute to most cancer-related deaths and circulating tumor cells (CTCs) have a pivotal role in metastatic relapse. CTCs are tumor cells that detach from the primary tumor and circulate in the bloodstream, subsequently metastasizing to distant organs. Measurement of CTCs by liquid biopsy can non-invasively provide reliable information regarding prognosis, recurrence, and treatment response compared to invasive diagnostic modalities such as transbronchial or fine-needle biopsy and thoracoscopic surgery.

CTCs include a variety of subtypes with different functional characteristics. Epithelial-mesenchymal transition (EMT), a process in which epithelial cells convert to mesenchymal cells, contributes substantively to tumor heterogeneity and is crucial to metastatic processes and the acquisition of chemo-resistance [1]. EMT typically involves the upregulation of mesenchymal markers such as vimentin and the downregulation of epithelial markers including EpCAM and cytokeratins [2]. Notably, recent studies support that EMT markers expressed on CTCs strongly associate with cancer metastasis in breast and hepatocellular carcinomas [3–5]. To date, various techniques have been proposed for isolating CTCs based on their physical features or cell surface antigens. Among these, the CellSearch™ System, which detects both EpCAM and cytokeratin expression, was the first and is the only clinically validated, FDA-cleared system for CTC identification [6, 7]. However, methods in which CTC capture is based on epithelial marker expression generally exhibit low sensitivity in patients with non-small cell lung cancer (NSCLC). The detection rate of the CellSearch™ system ranges from 36.4% to 75.7% in patients with advanced NSCLC [8, 9], and this technology has very low sensitivity in early-stage NSCLC [10]. Loss of epithelial markers results in an underestimation of CTC subpopulations that have undergone EMT in these conventional epithelial-based selection systems using anti-EpCAM and/or cytokeratin antibodies [1, 11].

Telomerase protects the chromosome ends from deterioration and fusion with neighboring chromosomes. It elongates the telomeres, which are short repeat sequences located at the end of the chromosomes. The telomere length determines the cell proliferation capacity and telomerase can render cells immortal through unlimited telomere lengthening. Whereas telomerase is not active in normal somatic cells, it may be so in cancer cells; more than 80% of cancer cells from various types of cancer are telomerase positive [12]. Thus, TelomeScan, an adenoviral vector (OBP-401) for cancer cell-specific GFP expression, uses telomerase as a cancer-specific marker for CTC detection. Specifically, TelomeScan can replicate only in cells in which the promoter for hTERT, a key enzyme of the telomerase complex, is activated.

Here we propose a new approach for the detection of CTCs including those with EMT status (EMT-CTCs) by using the new telomerase-specific replication-selective adenovirus, TelomeScan F35 (OPB-1101; rAdF35-142T-GFP) [13]. This technology allows for counting viable CTCs with very high sensitivity. TelomeScan F35 was further developed to suppress viral replication and GFP production in blood cells by targeting miR-142-3p, which is highly expressed only in blood cells [13]. miR-142-3p, which can prevent the transcription of downstream sequences including GFP, allowed accurate detection of CTCs, with a significant reduction in false-positive peripheral blood mononuclear cells (PBMCs) as compared to the conventional TelomeScan^®^ (OBP-401; rAd-GFP) [14]. To investigate the changes in phenotype of CTCs after detection by TelomeScan F35, immunocytochemistry was performed to evaluate CTCs with EMT status using both the epithelial marker EpCAM and the mesenchymal marker vimentin. We investigated the sensitivity of detection for CTCs and EMT-CTCs in NSCLC patients by TelomeScan F35 as a surrogate biomarker.

## RESULTS

### *In vitro* TelomeScan F35-based CTC detection assay validation in lung cancer cell lines

**Figure 1:**
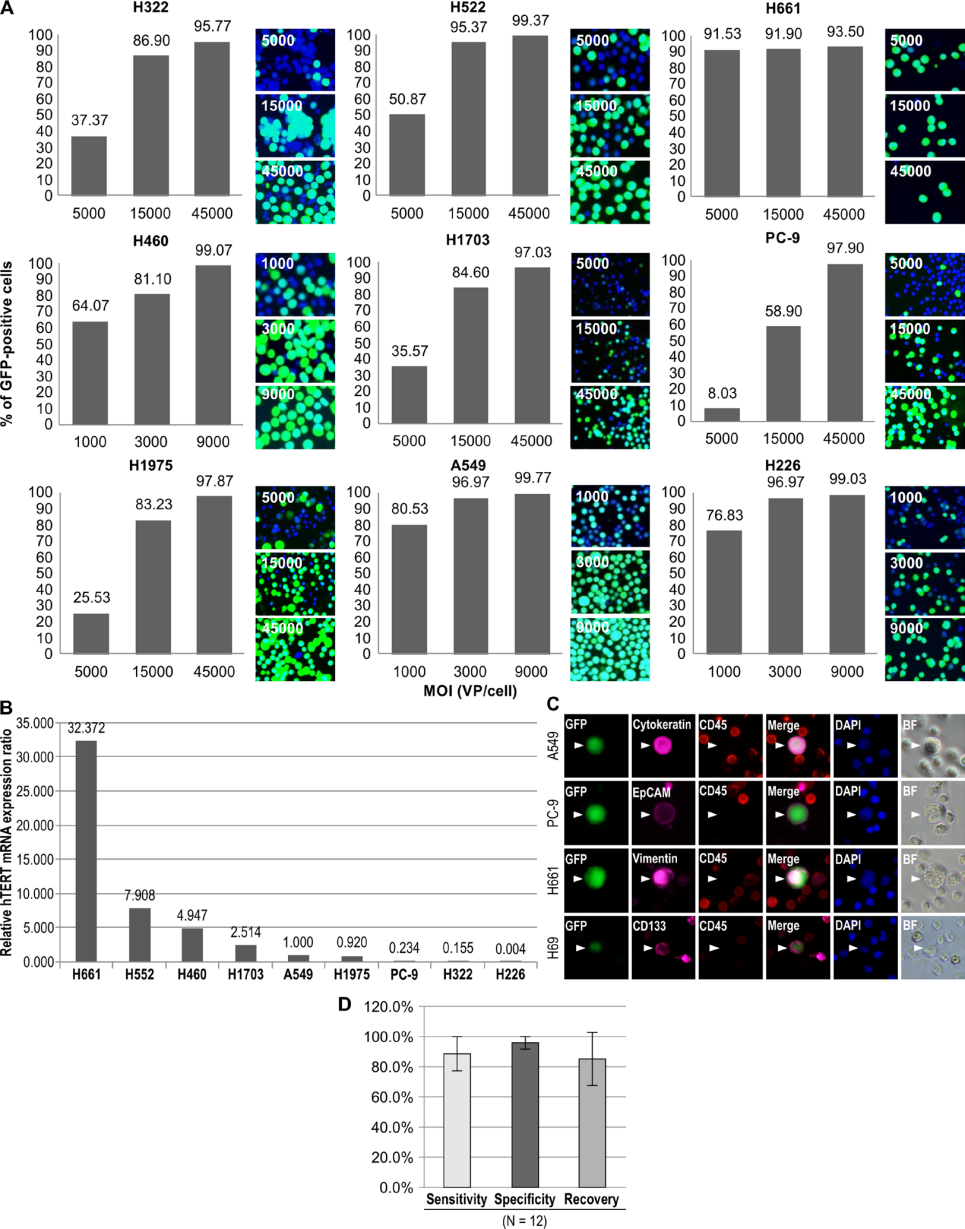
*In vitro* validation of the use of OBP-1101 for CTC detection using lung cancer cell lines with different hTERT expression levels The ratios of GFP+ cells in human NSCLC cell lines were determined by FACS analysis. (**A**) NSCLC cell lines were examined 24 h after inoculation of OBP-1101 at 1,000–45,000 VP/cell. Cell images were acquired under a fluorescence microscope. *hTERT* mRNA expression in human NSCLC cell lines was determined with qRT-PCR analysis. (**B**) *hTERT* mRNA expression was normalized to the expression in A549. (**C**) OBP-1101 could detect any type of lung cancer cells stained with epithelial (cytokeratin, EpCAM), mesenchymal (vimentin), or stem cell (CD133) markers. (**D**) For assay validation, we determined the sensitivity (GFP+ cells/marker+ cells), specificity (marker+ cells/GFP+ cells), and recovery (detected cells/spiked cells). To this end, 100 A549 cells were spiked into healthy blood and processed according to sample preparation methods. Cytokeratin was used as a cell marker.

We first investigated whether the infectivity of the TelomeScan F35 viral vector of cancer cells depended on hTERT activity. We performed quantitative reverse transcription (qRT)-PCR analysis to reveal the correlation between the rate of GFP+ cells and hTERT expression in various lung cancer cell lines. The hTERT expression level varied significantly among the lung cancer cell lines; however, the rate of GFP+ cells increased in a dose-dependent manner with multiplicity of infection (MOI; ranging from 1,000–45,000 virus particles (VP)/cell) in all lung cancer cell lines and was saturated at the highest MOI (Figure 1A, 1B).

Cells from lung cancer cell lines (A549, PC-9, H661, and H69) were spiked into 7.5 mL of blood from healthy volunteers as models of cancer patient blood. All examined lung cancer cell lines tested GFP+/CD45− using TelomeScan F35 and could further be identified by immunohistochemical staining of epithelial (cytokeratin, E-cadherin, or EpCAM), mesenchymal (vimentin), or cancer stem cell (CD133) markers (Figure 1C). As expected, the epithelial cancer cell lines were E-cadherin+/vimentin–whereas the mesenchymal cancer cell lines were E-cadherin−/vimentin+. The cancer stem cell marker CD133 was detected in GFP+ H69 cells.

To test the efficacy and accuracy of the assay, we determined the sensitivity, specificity, and recovery as the mean ratios of GFP-positive cells/cellular marker-positive cells, cellular marker-positive cells/GFP-positive cells, and detected cells/spiked cells, respectively. Whole-blood samples from healthy volunteers were spiked with 100 A549 cells and then analyzed. The sensitivity, specificity, and recovery were 89 ± 10%, 96 ± 4%, and 86 ± 18%, respectively, indicating high efficacy and accuracy of the assay system (Figure 1D).

### Detection of live CTCs in clinical samples from NSCLC patients

**Figure 2:**
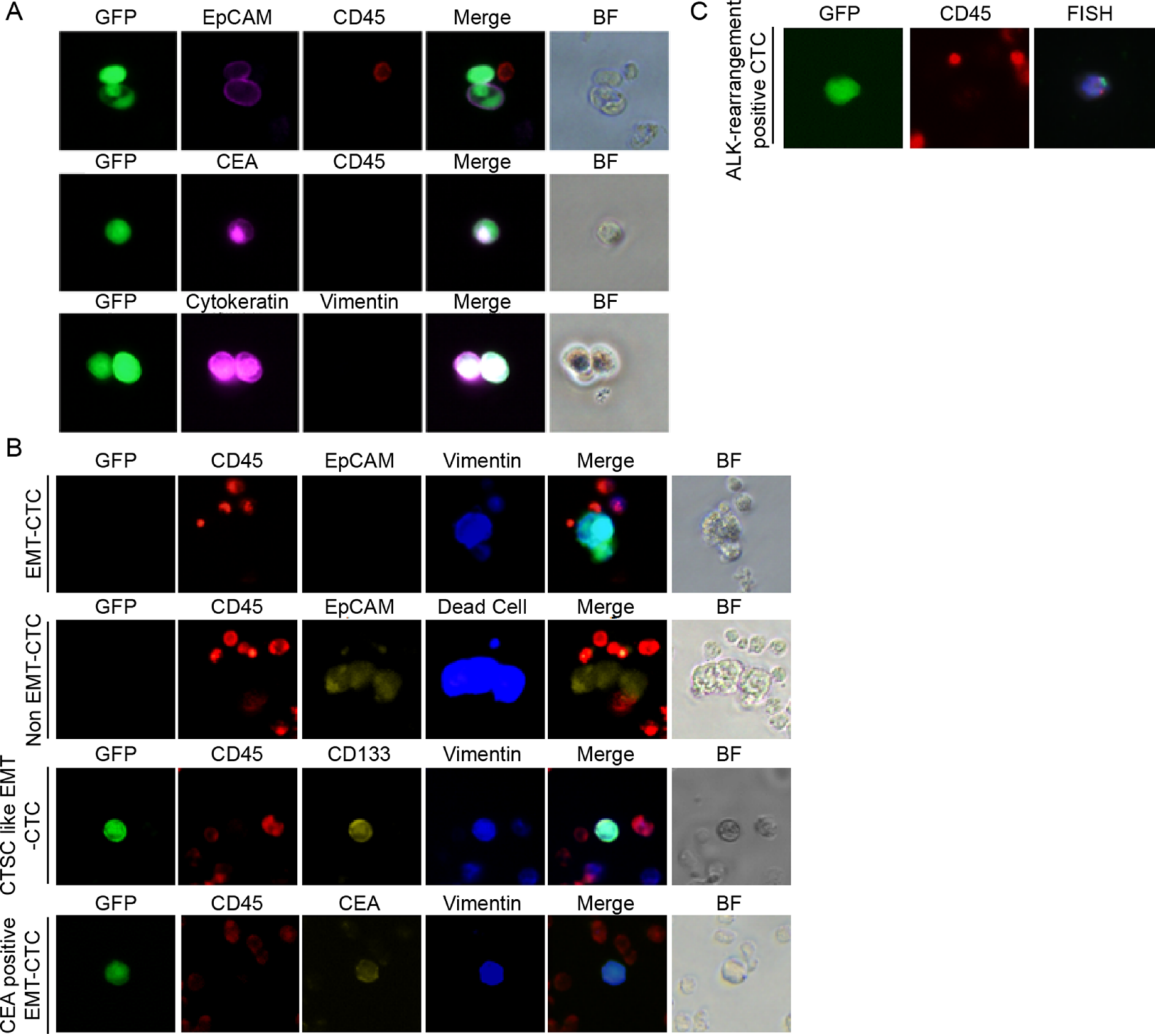
Viable CTC detection and phenotype characterization in NSCLC patients Cancer cells from lung cancer tissues were infected with OBP-1101 and characterized by immunostaining for cell markers. (**A**) Lung cancer cells in lavage solution. EpCAM and cytokeratin were used as epithelial markers, whereas vimentin and CEA were used as a mesenchymal and cancer marker, respectively. (**B**) Dead CTCs showing positive epithelial marker signal and viable CTCs showing mesenchymal marker signal. CTCs were detected by green fluorescence produced by OBP-1101 in NSCLC patients. These CTCs were viable because the virus can replicate only in viable cells. Additionally, these CTCs were classified as having a mesenchymal phenotype because they were stained by an antibody against vimentin, which is a typical mesenchymal cell marker. The epithelial albeit GFP-positive CTCs were detected by EpCAM staining and these epithelial CTCs were positive in live/dead staining. CD133 and CEA were positive in CTCs with vimentin positive detected by OBP-1101. (**C**) FISH analysis of GFP-positive cells. To show ALK-rearrangement, GFP-positive cells in blood samples from the metastatic NSCLC patients with confirmed ALK-rearrangement in tumors were subjected to FISH analysis. The nuclei present a split positive pattern with separation between the 5′ ALK green part and the 3′ ALK orange part of the FISH probe, which is consistent with ALK rearrangement.

We conducted a pilot study to evaluate the clinical feasibility of the detection system in 123 patients diagnosed with NSCLC. First, we inoculated lung cancer cells in lavage solution from surgically resected solid tumors with the TelomeScan F35 virus. TelomeScan F35 generated green fluorescence in cells that stained positive for monoclonal antibodies against markers including cytokeratin and CEA (Figure 2A).

We defined CTCs showing epithelial features and E-cadherin+/vimentin– staining as non-EMT-CTCs, and CTCs showing mesenchymal features and E-cadherin–/vimentin+ staining as EMT-CTCs. No synchronous CTCs that expressed dual staining as being both Vimentin and EpCAM positive were detected among the single CTCs. Human cancer cells undergoing EMT processes are considered to acquire cancer stemness characters and to exhibit potentiated metastatic abilities [15]. Accordingly, CD133 and CEA were detected in some GFP+/EpCAM– EMT-CTCs (Figure 2B). Epithelial but GFP– CTCs were detected in 20.8% of NSCLC patients by EpCAM staining. Notably, these epithelial CTCs were determined to be dead based on positive SYTOX Blue staining. Thus, TelomeScan F35 could specifically detect viable cancer cells including EMT-CTCs (Figure 2B). No clusters of viable CTCs (circulating tumor microemboli) were detected [7, 16] and all viable CTCs were present as single cells.

Some GFP-positive cells detected by the TelomeScan F35 in NSCLC patients with ALK-rearrangement were subjected to fluorescence *in situ* hybridization (FISH) analysis; the representative results are shown in Figure 2C. The nuclei of GFP-positive cells presented a split positive pattern with separation between the 5′ ALK green part and the 3′ ALK red part of the FISH probe, which is consistent with an ALK rearrangement.

### Prevalence of CTCs in NSCLC patients

CTCs were detected in 85/123 (69.1%) of patients. EMT-CTCs were detected in 57/123 (46.3%) of patients, whereas non-EMT-CTCs were detected in 48/123 (39.0%). Both EMT-CTCs and non-EMT-CTCs were detected in 20/123 (16.3%) of patients.

**Figure 3:**
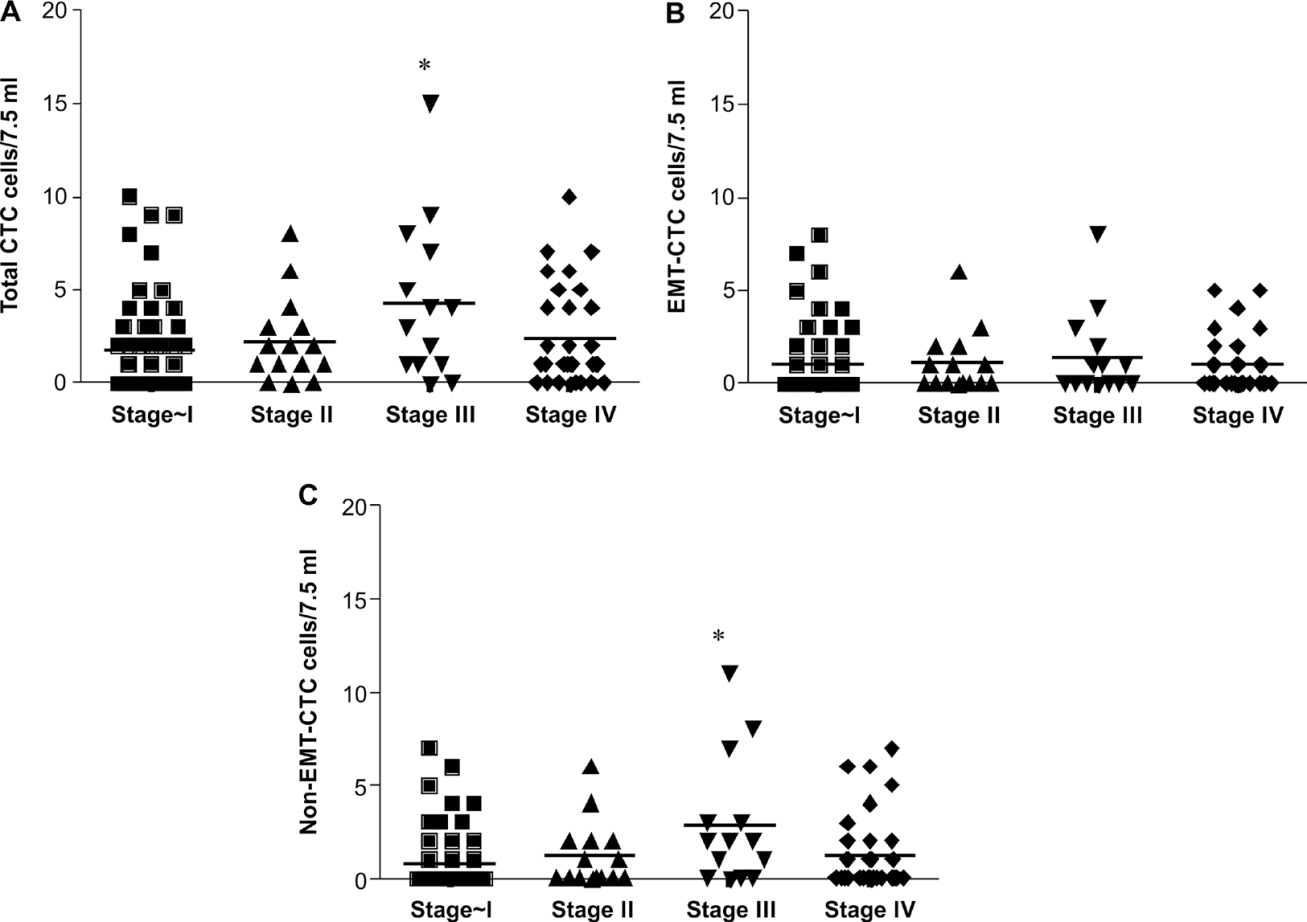
Distribution of CTC count in 7.5 mL peripheral blood from NSCLC patients according to stage (**A**) Total CTCs, (**B**) EMT-CTCs, (**C**) Non EMT-CTCs.

The CTC count did not correlate with disease stage (Figure 3). When 1 or more cells were GFP+/CD45– in the 7.5-mL peripheral blood sample, it was defined as positive (2.3 ± 0.3 cells/7.5 mL, range: 1–15 cells/7.5 mL) (Figure 3). However, the sensitivity of total CTC detection tended to be higher at the advanced stage (Table 1). The detection sensitivity was not significantly different between lung adenocarcinoma and squamous cell carcinoma cells (Table 1). The sensitivity of EMT-CTC detection slightly tended to increase in patients who showed pathological blood vessel invasion (*P* = 0.085); however, lymphovascular invasion and other histopathological classifications did not show significant differences in assay sensitivity (Supplementary Table 1). Pathologically, increased vessel invasion may be the reason for increased CTC release from primary tumors following the occurrence of EMT.

**Table 1:** Characteristics of patients and sensitivity of CTC according to final diagnosis

Sensitivity				
	(≥ 1 cell/7. 5 mL of peripheral blood tested positive)			
CTC phenotype	Total CTC	EMT-CTC	Non-EMT-CTC	EMT-CTC +Non-EMT-CTC
Primary NSCLC				
All patients (*n* = 123)	85 (69.1%)	57 (46.3%)	48 (39.0%)	20 (16.3%)
Histological type				
Adenocarcinoma (*n* = 100)	67 (67.0%)	43 (43.0%)	40 (40.0%)	15 (15.0%)
Squamous cell carcinoma (*n* = 19)	13 (68.4%)	10 (52.6%)	6 (31.6%)	3 (15.8%)
Others (*n* = 4)	4 (100%)	3 (75.0%)	2 (50.0%)	2 (50.0%)
Stage				
0 (*n* = 5)	2 (40.0%)	2 (40.0%)	0 (0%)	0 (0%)
I (*n* = 57)	35 (59.6%)	24(42.1%)	17 (24.6%)	6 (9.7%)
II (*n* = 15)	12 (40.0%)	7 (46.7%)	7 (46.7%)	2 (13.3%)
III (*n* = 14)	12 (85.7%)	7 (50.0%)	10 (71.4%)	5 (35.7%)
IV (*n* = 32)	24 (75.0%)	17 (53.1%)	14 (48.8%)	7 (21.9%)

### CTC detection for early diagnosis of NSCLC

As we could demonstrate high sensitivity of CTC detection by TelomeScan F35 in the early stage, TelomeScan F35 may be a useful tool for early diagnosis of NSCLC. To test this, we compared the detection sensitivity of TelomeScan F35 with that of the serum CEA, which is a popular tumor maker for early detection and screening of cancers, including lung cancer (Table 2). Patients with one or more CTCs were judged positive, whereas for serum CEA, two cut-off values were used. In early-stage (0–IA) lung adenocarcinoma patients, the TelomeScan F35-based detection sensitivity was 58.2%, whereas the sensitivity of CEA detection (cut-off value of 5.0 ng/mL) was only 11.7%. However, by combining both assays, we could achieve 65.1% sensitivity for the detection of early-stage NSCLC (CTC and CEA double-negative patients in stage 0–IA: 34.9%). Notably, 53.5% of early-stage patients tested negative in the CEA assay but positive for TelomeScan CTC detection (Table 2).

**Table 2:** Diagnostic values of CTC detection and serum CEA level at selected cut-off points in early-stage (0–IA) NSCLC patients

CEA > 5.0		Positive	Negative	
*n* = 43		Pts. (%)	Pts. (%)	*p*-value
Total CTC	(+)	2 (4.7%)	23 (53.5%)	
	(−)	3 (7.0%)	15 (34.9%)	*p* = 0.3818
CEA > 3.0		Positive	Negative	
*n* = 43		Pts. (%)	Pts. (%)	*p* value
Total CTC	(+)	8 (18.6%)	17 (39.5%)	
	(−)	8 (18.6%)	10 (23.3%)	*p* = 0.4049

### CTCs as surrogate markers of drug response

We analyzed whether changes in CTC detection and count according to the phenotype could predict the outcome of chemotherapy. To evaluate the relationship between CTC detection and drug response, tumors were categorized as either “responder” (complete response [CR] + partial response [PR]) or “non-responder” (stable disease [SD] + progressive disease [PD]) (Table 3). Only EMT-CTCs at baseline were significantly associated with poor drug response (*P* = 0.025). Detection of both EMT-CTCs and total CTCs was significantly negatively associated with prolonged PFS (EMT-CTC-positive vs. -negative: 193 ± 47 days vs. 388 ± 47 days, *P* = 0.040 and total CTC-positive vs. -negative: 231 ± 38 days vs. 393 ± 74 days, *P* = 0.042, respectively). In contrast, non-EMT-CTCs and total CTCs after 1 cycle of chemotherapy were not associated with drug response and prolonged progression-free survival (PFS).

**Table 3:** Correlation between CTC detection/count and treatment response

		Responder	Non-responder	Pearson		PFS (days)	Student *t*
		Pts. (%)	Pts. (%)	*p*-value	Pts. (%)	Mean ± SE	*P*-value
EMT-CTC	(+)	3 (10.0%)	12 (40.0%)		14 (50.0%)	193.1 ± 47.4	
	(−)	9 (30.0%)	6 (20.0%)	0.0253	14 (50.0%)	338.1 ± 47.4	0.0402
Non EMT-CTC	(+)	10 (33.3%)	9 (30.0%)		17 (60.7%)	267.8 ± 45.4	
	(−)	2 (6.67%)	9 (30.0%)	0.0634	11 (39.3%)	262.2 ± 60.7	0.9414
Total CTC	(+)	10 (33.3%)	14 (46.7%)		22 (78.6%)	231.0 ± 38.4	
	(−)	2 (6.67%)	4 (13.3%)	0.7094	6 (21.4%)	392.7 ± 73.5	0.0415
Total CTC	(+)	3 (27.3%)	3 (27.3%)		6 (54.5%)	291.2 ± 77.2	
(After 1 cycle)	(−)	2 (18.2%)	3 (27.3%)	0.7401	5 (45.5%)	322.2 ± 84.5	0.7933

Notably, only the EMT-CTC count at baseline was significantly associated with drug response as indicated by logistic regression analysis (*P* = 0.017) (Supplementary Figure 1) and showed a negative correlation with PFS (*P* = 0.010) (Supplementary Figure 2). Changes in CTC count after 1 cycle of chemotherapy were measured (*n* = 11). The total CTC count decreased in 5 out of 11 (45.5%) patients, whereas 3 out of these 5 patients did not show a response. Moreover, 2 out of 4 patients showed a response even though the CTC number increased after 1 cycle of chemotherapy. Similarly, the non-EMT-CTC count did not predict treatment response before and after administration of 1 cycle of chemotherapy. Overall, four out of 5 (80.0%) patients did not respond to therapy although EMT-CTCs decreased after 1 cycle of chemotherapy in these patients. Notably, all 5 patients who had no detectable EMT-CTCs at baseline showed a positive response to treatment. Taken together, our results indicated that patients positive for EMT-CTC at baseline demonstrated poor clinical response to anticancer drugs (Supplementary Figure 3).

## DISCUSSION

In the present study, we validated the TelomeScan F35 *in vitro* assay system for CTC detection using lung cancer cell lines with different phenotypes and varying hTERT expression that were spiked in blood from healthy volunteers, revealing close to 90% sensitivity, specificity, and recovery. TelomeScan F35 was found to be a highly sensitive detection system for viable CTCs in blood samples when compared to the serum CEA level in patients with early-stage (0–IA) NSCLC, and thus, might provide a useful screening tool for early diagnosis. We demonstrated that CTCs with mesenchymal phenotype (EMT-CTCs) were common in all stages. Notably, EMT-CTCs existed in early-stage NSCLC patients, even before surgical resection. The presence of EMT-CTCs predicted poor clinical outcome and is a potential surrogate indicator of chemotherapeutic efficacy in NSCLC patients.

Even after complete surgical resection, 30% of patients diagnosed with stage I NSCLC show tumor recurrence and die [17, 18]. T*he existence of* micrometastatic tumor cells in distant organs and peripheral blood has been reported [19–23]. NSCLC patients having occult micrometastatic cancer cells in the lymph nodes were associated with a poor prognosis, even at the early stage I [23]. EMT generates cells that are able to invade the blood stream [24] from tumor tissue and it has been suggested that CTCs undergo EMT in order to migrate to distant organs [25]. NSCLC or breast cancer patients testing CTC-positive at the time of definitive surgery showed short recurrence periods [10, 26, 27]. Recently, CTCs were detected one month after surgery in patients who underwent radical resection for NSCLC, and the presence of CTCs was significantly associated with early recurrence and shorter disease-free survival [28]. We could detect EMT-CTCs with high sensitivity in resected NSCLC patients, even at the early stage. Moreover, patients with detectable levels of EMT-CTC before complete resection may have a higher risk of relapse. Thus, EMT-CTC detection may be a reliable biomarker for use in decision making regarding adjuvant chemotherapy targeting EMT-CTCs following curative resection in future clinical studies. CTCs were detected in patients with various pathological types including pleomorphic carcinomas with NSCLC components known to be associated with rapid relapse and histologically poor differentiation, and the EMT-CTCs tended to be higher in NSCLC patients with pathological vessel invasion. Our results indicate that EMT-CTCs may provide a reliable biomarker to predict recurrence-free survival after curative resection in combination with information on histopathological background from resected tumor tissues.

In resected primary tumors, increased expression of EMT markers TWIST1 and Snail has been observed in stage I NSCLC [29, 30]. Overexpression of the EMT markers in stage I NSCLC was associated with worse overall survival and shorter recurrence-free survival. Furthermore, monitoring of CTC-positive chronic obstructive pulmonary disease patients with clinically undetectable lung cancer by CT-scan screening allowed detecting early-stage lung cancer, which showed an EMT marker phenotype similar to that of the CTCs [31]. These reports support the notion that the appearance of EMT-CTCs may be a sensitive biomarker for early diagnosis, even in the case of clinically undetectable lung cancer. Notably, the present study demonstrated that measurement of CTCs using TelomeScan F35 allowed the detection of EMT-CTCs from stage 0 in patients with NSCLC pathologically diagnosed as adenocarcinoma *in situ*.

Among blood-based biomarkers, serum CEA is the most extensively examined, especially in adenocarcinoma of the lung, to determine tumor progression and prognosis. However, routine use of serum CEA in clinical practice is not recommended mainly because of insufficient sensitivity and specificity. Thus, we assessed the diagnostic performance of CTC count in comparison with that of serum CEA in the present study. A large fraction (53.5%) of early-stage (stages 0–IA) patients detected to be CTC-positive tested CEA-negative. Our results indicate that CTC detection by TelomeScan F35 could be a useful non-invasive screening tool for the early diagnosis of NSCLC in patients who cannot be diagnosed on the basis of current clinical biomarkers but only via invasive diagnostic modalities including transbronchial or fine-needle biopsy and thoracoscopic surgery because of small primary lesions.

Lung cancer patients exhibiting high CTC counts at baseline are associated with poor prognosis [7, 16]. Some previous studies have indicated that the clinical stage does not correlate with CTC count [32–34]. Similarly, the number of CTCs detected by TelomeScan F35 did not show a clear correlation with staging, although the sensitivity of CTC detection tended to be higher in the advanced than in the early stage. Thus, it remains unclear whether the total CTC count associates with staging. The EMT-CTC population increased when patients acquired drug resistance and developed recurrence [1]. Increased staining of CTCs with CD133, a lung cancer stem cell marker, or the presence of EMT-CTCs was significantly associated with shortened PFS in NSCLC patients who were administrated platinum-based chemotherapy [35]. We observed that NSCLC patients who tested positive for the presence of EMT-CTCs with some CD133+ staining before treatment showed short PFS, although for some patients, EMT-CTCs went from positive at baseline to negative after 1 cycle of chemotherapy. Thus, detection of CD133+ EMT-CTCs and positive EMT-CTC assay results at baseline predicted poor clinical outcome. Our results support the previous studies and suggest that the population of CTCs with mesenchymal phenotype, rather than total CTC count, potentially represents a sensitive surrogate biomarker.

However, in breast cancer patients with persistently increased CTCs as determined with the CellSearch™ System, which detects only non-EMT-CTCs, early switching to an alternate cytotoxic therapy after 1 cycle of first-line chemotherapy failed to prolong overall survival in a randomized trial (SWOG S0500) [36]. Alternatively, detecting the presence of the echinoderm microtubule-associated protein-like 4 (EML4)-anaplastic lymphoma kinase (ALK) fusion gene, termed the ALK-rearrangement, is routinely performed in lung adenocarcinomas and allows the treatment of such patients with anti-ALK targeted therapy. FISH assays for the detection of CTCs with ALK-rearrangements can therefore provide additional valuable information that may be clinically helpful for treatment planning [37]. Thus, further studies are warranted to provide molecular evidence of the predictive value of CTCs for early diagnosis of recurrence in patients with lung cancer under various treatment regimens. However, our findings indicate that CTCs, especially, EMT-CTCs, can be expected to be more reliable markers for drug response and prognosis.

In conclusion, TelomeScan F35 achieved high sensitivity for viable CTCs, even in patients with early-stage NSCLC. Unlike the EpCAM selection system, TelomeScan F35 allows detecting CTCs with mesenchymal phenotype. We observed that EMT-CTCs were quite common in NSCLC. Because EMT determines the metastatic potential and the acquisition of therapeutic resistance, CTCs with the mesenchymal phenotype may be a more reliable biomarker for predicting clinical outcome in lung cancer patients and a potential surrogate indicator of chemotherapeutic efficacy in NSCLC patients. In the present study, the small sample number was a limitation; accordingly, the clinical significance of CTC detection using our highly sensitive TelomeScan F35 detection system according to the CTC phenotype should be independently validated in “*at risk*” patients in larger clinical prospective studies. Finally, the predictive value of EMT-CTCs as biomarkers for prognosis, relapse, and drug response need to be further clarified in future studies.

## MATERIALS AND METHODS

### Cell culture

The human NSCLC cell lines H661, A549, H522, H322, H226, H1975, H1703, and H460 and the human SCLC cell line H69 were purchased from American Type Culture Collection (Manassas, VA). PC-9 was provided by the RIKEN BRC through the National Bio-Resource Project of the MEXT, Japan. All cell lines were cultured with Dulbecco's modified Eagle's medium (DMEM) supplemented with 10% FCS, 100 μg/mL penicillin, and 250 μg/mL streptomycin in a humidified atmosphere of 5% CO_2_.

### FACS analysis

Cells were trypsinized with 0.25% trypsin-EDTA (Cat No. 4049-500ML, Sigma-Aldrich, St. Louis, MO) and fixed with 4% paraformaldehyde following 24-h inoculation of OBP-1101 at 1,000–45,000 VP/cell. Fixed cells were analyzed using a MACSQuant^®^ Analyzer (Miltenyi Biotec, Bergisch-Gladbach, Germany) to determine the ratio of GFP+ cells.

### qRT-PCR analysis

Total RNA was extracted from human NSCLC cell lines using the RNeasy Plus Mini Kit (Cat No. 74134, Qiagen, Venlo, The Netherlands). qRT-PCR was performed using the PrimeScript RT reagent kit (TaKaRa, Shiga, Japan) and SYBR Premix EX Taq II (Tli RNaseH Plus, TaKaRa) on a 7500 Fast real-time PCR system (Applied Biosystems, Foster City, CA). The following gene-specific primers were used: *hTERT* F (5′-CGG AAG AGT GTC TGG AGC AA-3′), R (5′-GGA TGA AGC GGA GTC TGG A-3′), *GAPDH* F (5′-TCG ACA GTC AGC CGC ATC TTC TTT-3′), R (5′-ACC AAA TCC GTT GAC TCC GAC CTT-3′). Thermal cycling conditions were as follows: 95°C for 1 min followed by 45 cycles of 95°C for 10 s, 57°C for 30 s, and 72°C for 30 s. *GAPDH* was used as an internal control [38].

### Patient characteristics

Peripheral venous blood samples were obtained from 123 patients who were pathologically diagnosed as having primary NSCLC at Juntendo University Hospital from July 2013 to December 2015. The procedures for obtaining peripheral blood from patients with lung cancer were approved by the Institutional Review Board at the Juntendo University School of Medicine (No. 2013067). All patients and volunteers provided written informed consent. Thoracotomy was performed in 82 primary lung cancer patients who were all p-stage 0-IIIA. In addition, 9 patients with c-stage III disease and 32 patients with c-stage IV disease were diagnosed without thoracotomy (Table 1). Overall, 100 patients had adenocarcinoma and 19 patients had squamous cell carcinoma. Of the remaining 4 patients, 3 had pleomorphic carcinoma with adenocarcinoma component and 1 had large cell carcinoma. For 30 patients with relapsed or advanced NSCLC who received chemotherapy at Juntendo University Hospital, CTCs were measured within 2 weeks before starting standard chemotherapy. For 11 out of these 30 patients, CTCs were also measured after the first cycle of first-line chemotherapy. PFS was determined as the duration between the first day of standard chemotherapy and the date of objective disease progression. Response Evaluation Criteria in Solid Tumors (ver. 1.1) was applied for the evaluation of tumor response using computed tomography.

### Sample preparation and viral infection

Briefly, 7.5-mL blood samples were collected from patients who were histologically diagnosed with NSCLC and healthy volunteers and sent to Clinical Laboratory Center, Oncolys BioPharma Inc. for analysis within one day. The blood samples were processed with red blood cell lysis buffer containing ammonium chloride (NH_4_Cl) (pH 7.3, custom-made, Wako Pure Chemical Industries, Osaka, Japan) for 5 min followed by centrifugation at 300 × *g* for 5 min at 25°C to collect the white blood cells (WBCs). Isolated WBCs were washed with DMEM containing 10% fetal bovine serum (FBS, Cat No. 10437-028, Gibco, Gaithersburg, MD) twice (first in 12 mL and then in 5 mL). The cell pellet was transferred to a 1.5-mL tube and incubated with 1 × 10^9^ VP of OBP-1101 (rAdF35-142T-GFP) in 1 mL of DMEM containing 10% FBS at 37°C on a rotator (WKN-2210, Waken Btech, Kyoto, Japan) for 24 h. No GFP+ PBMCs were detected from healthy donors (*n* = 17) following inoculation with 1 × 10^9^ VP indicating that this amount was suitable to accurately detect CTCs without producing false-positive cells in patients (13). For this reason, the presence of more than 1 CTC in 7.5 mL of blood was defined as positive in this study. The OBP-1101 used in this study was from Oncolys BioPharma Inc. (OBP-1101, lot no. γ-1, Tokyo, Japan). The study protocols described above were approved by the institutional review boards of Juntendo University and Kenshokai Fukushima Healthcare Center, which is the affiliated institution of Oncolys BioPharma Inc.

### Immunofluorescence staining

Cells were immunostained with primary antibodies at room temperature for 30 min following fixation and permeabilization with 4% paraformaldehyde (PFA) (Cat No. 09154-85, Nacalai-Tesque, Kyoto, Japan) and 0.15% Triton X-100, (Cat No. 93343-100ML, Sigma) respectively. Primary antibodies used were anti-CD45 (Cat No.304002, 1:100 or 304032, 3:100; BioLegend, San Diego, CA), anti-CD45 conjugated with Brilliant Violet 421 (304032, 3:100; BioLegend), anti-EpCAM (ab7504, 1:100; Abcam), anti-CD133 (130-090-422, 1:20; Miltenyi Biotec), anti-vimentin (ab45939, 1:200; Abcam, Cambridge, UK), anti-pan-cytokeratin (628602, 1:100; BioLegend), anti-cytokeratin 19 (628502, 1:100; BioLegend), and anti-CEA (M707229, 1:50; Dako Corp., Carpenteria, CA). For signal amplification, secondary antibodies (A21422, A21235, A11046, A21245, 1:200; Invitrogen, Carlsbad, CA) or a labeling kit (Z25005, Invitrogen) were used. Nuclear DNA was stained with SYTOX Blue (Molecular Probes, Eugene, OR) to confirm the viability of the CTCs. Cell surface markers were immunostained before fixation.

### FISH for GFP-positive cells

Cells infected with OBP-1101 were blocked with 10% FBS and 0.02 μg/μL DNase (Cat No. 11284932001, Roche, Madison, WI) in PBS (Cat No. 164-25511, Wako) and incubated with an anti-human CD45 antibody (1:200 dilution) at room temperature for 30 min. Cells were washed with washing buffer (2% FBS in PBS) and incubated with Alexa Fluor 647 goat anti-mouse IgG (1:200 dilution; Cat No. A21235, Life Technologies, Carlsbad, CA) at room temperature for 30 min, washed, and fixed with 4% PFA in PBS. CTCs were collected on slide glass (Cat No. S9441, Matsunami, Bellingham, WA) using a PicoPipet system (NEPAGENE, Ichikawa, Japan), air dried, and dehydrated by EtOH (Cat No. 057-00456, Wako). To demonstrate the ALK rearrangement in nuclei of GFP-positive cells, FISH was performed using the Food and Drug Administration-approved FISH test (Vysis LSI *ALK* Dual Color Break Apart FISH probe, Abbott Molecular, Inc., Abbott Park, IL) with a Abbott Histology FISH Accessory kit (Vysis Paraffin & Post-Hybridization Wash Buffer kit, Abbott Molecular, Inc.) following the manufacturer's instructions.

### Fluorescence microscopy for CTC detection

Immunostained cells were resuspended in 2% FBS/PBS and transferred into 96-well plates. GFP+ or marker+ cells were detected by fluorescence microscopy (Eclipse Ti, Nikon) at a magnification of 20×. Cell images were acquired on a Metamorph (Molecular Devices, Sunnyvale, CA) using each filter set (DAPI/FITC/RFP/CY5, Olympus, Tokyo, Japan). GFP+ CTC and false-positive cells were discriminated based on their anti-CD45 staining status as follows: CTC (GFP+/CD45−) and false positive (GFP+/CD45+). Fluorescence intensity of anti-CD45 staining was quantified from acquired cell images using the NIS-elements software (Nikon, Tokyo, Japan).

### Statistical analysis

The association of the number of CTCs with clinical characteristics, serum carcinoembryonic antigen (CEA) level, and treatment response were evaluated using Pearson's Chi-squared test for categorical variables as appropriate. The association of the number of CTCs with treatment response was analyzed using Spearman rank correlation and logistic regression analysis, and the Wilcoxon test was used for comparing groups before and after treatment. The Mann-Whitney *U* test was used for comparisons between two groups and the Kruskal-Wallis test was used for comparisons among three or more groups if the sample distribution was asymmetrical. For each test, *P*-values < 0.05 were considered statistically significant. All statistical analyses were done using the JMP 10.0 software for Windows.

## SUPPLEMENTARY MATERIALS FIGURES AND TABLES



## References

[1] Yu M, Bardia A, Wittner BS, Stott SL, Smas ME, Ting DT, Isakoff SJ, Ciciliano JC, Wells MN, Shah AM, Concannon KF, Donaldson MC, Seguist LV (2013). Circulating breast tumor cells exhibit dynamic changes in epithelial and mesenchymal composition. Science.

[2] Thiery JP (2002). Epithelial-mesenchymal transitions in tumour progression. Nat Rev Cancer.

[3] Kallergi G, Papadaki MA, Politaki E, Mavroudis D, Georgoulias V, Agelaki S (2011). Epithelial to mesenchymal transition markers expressed in circulating tumour cells of early and metastatic breast cancer patients. Breast Cancer Res.

[4] Li YM, Xu SC, Li J, Han KQ, Pi HF, Zheng L, Zuo GH, Huang XB, Li HY, Zhao HZ, Yu ZP, Zhou Z, Liang P (2013). Epithelial-mesenchymal transition markers expressed in circulating tumor cells in hepatocellular carcinoma patients with different stages of disease. Cell Death Dis.

[5] Wu S, Liu S, Liu Z, Huang J, Pu X, Li J, Yang D, Deng H, Yang N, Xu J (2015). Classification of circulating tumor cells by epithelial-mesenchymal transition markers. PloS ONE.

[6] Cristofanilli M, Hayes DF, Budd GT, Ellis MJ, Stopeck A, Reuben JM, Doyle GV, Matera J, Allard WJ, Miller MC, Fritsche HA, Hortobagyi GN, Terstappen LW (2005). Circulating tumor cells: a novel prognostic factor for newly diagnosed metastatic breast cancer. J Clin Oncol.

[7] Krebs MG, Sloane R, Priest L, Lancashire L, Hou JM, Greystoke A, Ward TH, Ferraldeschi R, Hughes A, Clack G, Ranson M, Dive C, Blackhall FH (2011). Evaluation and prognostic significance of circulating tumor cells in patients with non-small-cell lung cancer. J Clin Oncol.

[8] Hirose T, Murata Y, Oki Y, Sugiyama T, Kusumoto S, Ishida H, Shirai T, Nakashima M, Yamaoka T, Okuda K, Ohnishi T, Ohmori T (2012). Relationship of circulating tumor cells to the effectiveness of cytotoxic chemotherapy in patients with metastatic non-small-cell lung cancer. Oncol Res.

[9] Punnoose EA, Atwal S, Liu W, Raja R, Fine BM, Hughes BG, Hicks RJ, Hampton GM, Amler LC, Pirzkall A, Lackner MR (2012). Evaluation of circulating tumor cells and circulating tumor DNA in non-small cell lung cancer: association with clinical endpoints in a phase II clinical trial of pertuzumab and erlotinib. Clin Cancer Res.

[10] Sawabata N, Okumura M, Utsumi T, Inoue M, Shiono H, Minami M, Nishida T, Sawa Y (2007). Circulating tumor cells in peripheral blood caused by surgical manipulation of non-small-cell lung cancer: pilot study using an immunocytology method. Gen Thorac Cardiovasc Surg.

[11] Gorges TM, Tinhofer I, Drosch M, Rose L, Zollner TM, Krahn T, von Ahsen O (2012). Circulating tumour cells escape from EpCAM-based detection due to epithelial-to-mesenchymal transition. BMC Cancer.

[12] Kim NW, Piatyszek MA, Prowse KR, Harley CB, West MD, Ho PL, Coviello GM, Wright WE, Weinrich SL, Shay JW (1994). Specific association of human telomerase activity with immortal cells and cancer. Science.

[13] Sakurai F, Narii N, Tomita K, Togo S, Takahashi K, Machitani M, Tachibana M, Ouchi M, Katagiri N, Urata Y, Fujiwara T, Mizuguchi H (2016). Efficient detection of human circulating tumor cells without significant production of false-positive cells by a novel conditionally replicating adenovirus. Mol Ther Methods Clin Dev.

[14] Kojima T, Hashimoto Y, Watanabe Y, Kagawa S, Uno F, Kuroda S, Tazawa H, Kyo S, Mizuguchi H, Urata Y, Tanaka N, Fujiwara T (2009). A simple biological imaging system for detecting viable human circulating tumor cells. J Clin Invest.

[15] Mani SA, Guo W, Liao MJ, Eaton EN, Ayyanan A, Zhou AY, Brooks M, Reinhard F, Zhang CC, Shipitsin M, Campbell LL, Polyak K, Brisken C (2008). The epithelial-mesenchymal transition generates cells with properties of stem cells. Cell.

[16] Hou JM, Krebs MG, Lancashire L, Sloane R, Backen A, Swain RK, Priest LJ, Greystoke A, Zhou C, Morris K, Ward T, Blackhall FH, Dive C (2012). Clinical significance and molecular characteristics of circulating tumor cells and circulating tumor microemboli in patients with small-cell lung cancer. J Clin Oncol.

[17] Martini N, Bains MS, Burt ME, Zakowski MF, McCormack P, Rusch VW, Ginsberg RJ (1995). Incidence of local recurrence and second primary tumors in resected stage I lung cancer. J Thorac Cardiovasc Surg.

[18] Nesbitt JC, Putnam JB, Walsh GL, Roth JA, Mountain CF (1995). Survival in early-stage non-small cell lung cancer. Ann Thorac Surg.

[19] Chen ZL, Perez S, Holmes EC, Wang HJ, Coulson WF, Wen DR, Cochran AJ (1993). Frequency and distribution of occult micrometastases in lymph nodes of patients with non-small-cell lung carcinoma. J Natl Cancer Inst.

[20] Pantel K, Izbicki JR, Angstwurm M, Braun S, Passlick B, Karg O, Thetter O, Riethmüller G (1993). Immunocytological detection of bone marrow micrometastasis in operable non-small cell lung cancer. Cancer Res.

[21] Pantel K, Izbicki J, Passlick B, Angstwurm M, Haussinger K, Thetter O, Riethmüller G (1996). Frequency and prognostic significance of isolated tumour cells in bone marrow of patients with non-small-cell lung cancer without overt metastases. Lancet.

[22] Hashimoto T, Kobayashi Y, Ishikawa Y, Tsuchiya S, Okumura S, Nakagawa K, Tokuchi Y, Hayashi M, Nishida K, Hayashi S, Hayashi J, Tsuchiya E (2000). Prognostic value of genetically diagnosed lymph node micrometastasis in non-small cell lung carcinoma cases. Cancer Res.

[23] Osaki T, Oyama T, Gu CD, Yamashita T, So T, Takenoyama M, Sugio K, Yasumoto K (2002). Prognostic impact of micrometastatic tumor cells in the lymph nodes and bone marrow of patients with completely resected stage I non-small-cell lung cancer. J Clin Oncol.

[24] Kalluri R, Weinberg RA (2009). The basics of epithelial-mesenchymal transition. J Clin Invest.

[25] Aktas B, Tewes M, Fehm T, Hauch S, Kimmig R, Kasimir-Bauer S (2009). Stem cell and epithelial-mesenchymal transition markers are frequently overexpressed in circulating tumor cells of metastatic breast cancer patients. Breast Cancer Res.

[26] Rack B, Schindlbeck C, Juckstock J, Andergassen U, Hepp P, Zwingers T, Friedl TW, Lorenz R, Tesch H, Fasching PA, Fehm T, Schneeweiss A, Lichtenegger W, SUCCESS Study Group (2014). Circulating tumor cells predict survival in early average-to-high risk breast cancer patients. J Natl Cancer Inst.

[27] Lucci A, Hall CS, Lodhi AK, Bhattacharyya A, Anderson AE, Xiao L, Bedrosian I, Kuerer HM, Krishnamurthy S (2012). Circulating tumour cells in non-metastatic breast cancer: a prospective study. Lancet Oncol.

[28] Bayarri-Lara C, Ortega FG, Cueto Ladron de Guevara A, Puche JL, Ruiz Zafra J, de Miguel-Perez D, Ramos AS, Giraldo-Ospina CF, Navajas Gómez JA, Delgado-Rodriguez M, Lorente JA, Serrano MJ (2016). Circulating tumor cells identify early recurrence in patients with non-small cell lung cancer undergoing radical resection. PloS ONE.

[29] Hung JJ, Yang MH, Hsu HS, Hsu WH, Liu JS, Wu KJ (2009). Prognostic significance of hypoxia-inducible factor-1α, TWIST1 and Snail expression in resectable non-small cell lung cancer. Thorax.

[30] Jiang W, Pang XG, Wang Q, Shen YX, Chen XK, Xi JJ (2012). Prognostic role of Twist, Slug, and Foxc2 expression in stage I non-small-cell lung cancer after curative resection. Clin Lung Cancer.

[31] Ilie M, Hofman V, Long-Mira E, Selva E, Vignaud JM, Padovani B, Mouroux J, Marguette CH, Hofman P (2014). circulating tumor cells allow early diagnosis of lung cancer in patients with chronic obstructive pulmonary disease. PloS ONE.

[32] Ge M, Shi D, Wu Q, Wang M, Li L (2005). Fluctuation of circulating tumor cells in patients with lung cancer by real-time fluorescent quantitative-PCR approach before and after radiotherapy. J Cancer Res Ther.

[33] Yoon SO, Kim YT, Jung KC, Jeon YK, Kim BH, Kim CW (2011). TTF-1 mRNA-positive circulating tumor cells in the peripheral blood predict poor prognosis in surgically resected non-small cell lung cancer patients. Lung Cancer.

[34] Wendel M, Bazhenova L, Boshuizen R, Kolatkar A, Honnatti M, Cho EH, Marrinucci D, Sandhu A, Perricone A, Thistlethwaite P, Bethel K, Nieva J, van den Heuvel M (2012). Fluid biopsy for circulating tumor cell identification in patients with early-and late-stage non-small cell lung cancer: a glimpse into lung cancer biology. Phys Biol.

[35] Nel I, Jehn U, Gauler T, Hoffmann AC (2014). Individual profiling of circulating tumor cell composition in patients with non-small cell lung cancer receiving platinum based treatment. Translat Lung Cancer Res.

[36] Smerage JB, Barlow WE, Hortobagyi GN, Winer EP, Leyland-Jones B, Srkalovic G, Tejwani S, Schott AF, O’Rourke MA, Lew DL, Doyle GV, Gralow JR, Livingston RB (2014). Circulating tumor cells and response to chemotherapy in metastatic breast cancer: SWOG S0500. J Clin Oncol.

[37] Soda M, Choi YL, Enomoto M, Takada S, Yamashita Y, Ishikawa S, Fujiwara S, Watanabe H, Kurashina K, Hatanaka H, Bando M, Ohno S, Ishikawa Y (2007). Identification of the transforming EML4-ALK fusion gene in non-small-cell lung cancer. Nature.

[38] Terrin L, Trentin L, Degan M, Corradini I, Bertorelle R, Carli P, Maschio N, Bo MD, Noventa F, Gattei V, Semenzato G, de Rossi A (2007). Telomerase expression in B-cell chronic lymphocytic leukemia predicts survival and delineates subgroups of patients with the same igVH mutation status and different outcome. Leukemia.

