# Supplementary material for: Sensitive detection of viable circulating tumor cells using a novel conditionally telomerase-selective replicating adenovirus in non-small cell lung cancer patients
Source: Oncotarget. 2017 Apr 4;8(21):34884–95. doi: 10.18632/oncotarget.16818 (PMC5471019; doi:10.18632/oncotarget.16818)
Supplement: Supplementary file 1 [file oncotarget-08-34884-s001.pdf]

Sensitive detection of viable circulating tumor cells using a novel conditionally telomerase-selective replicating adenovirus in non-small cell lung cancer patients

Supplementary Materials

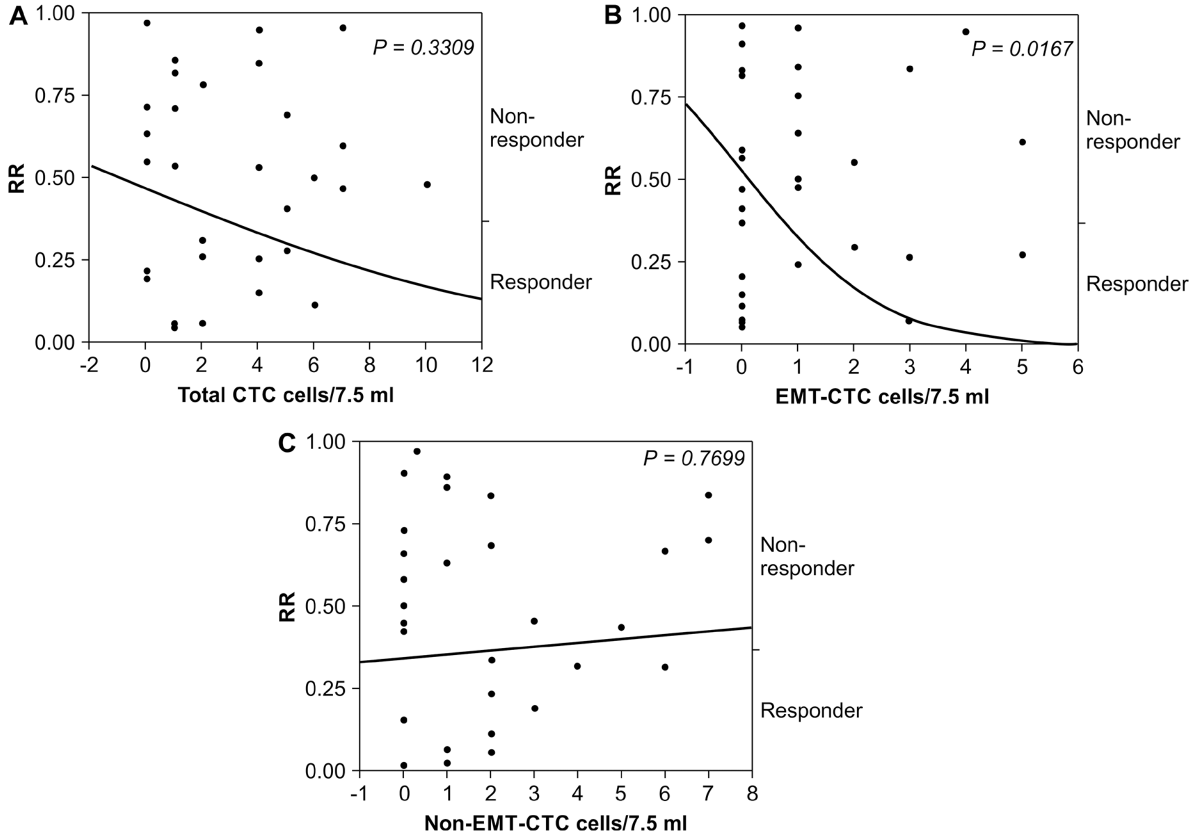

Supplementary Figure 1: CTC count in 7.5 mL of peripheral blood from NSCLC patients for prediction for drug response (*n* = 30). Drug response was categorized as either responder (CR+PR) or non-responder (SD+PD). (**A**) Total CTCs, (**B**) EMT-CTCs, (**C**) Non EMT-CTCs.

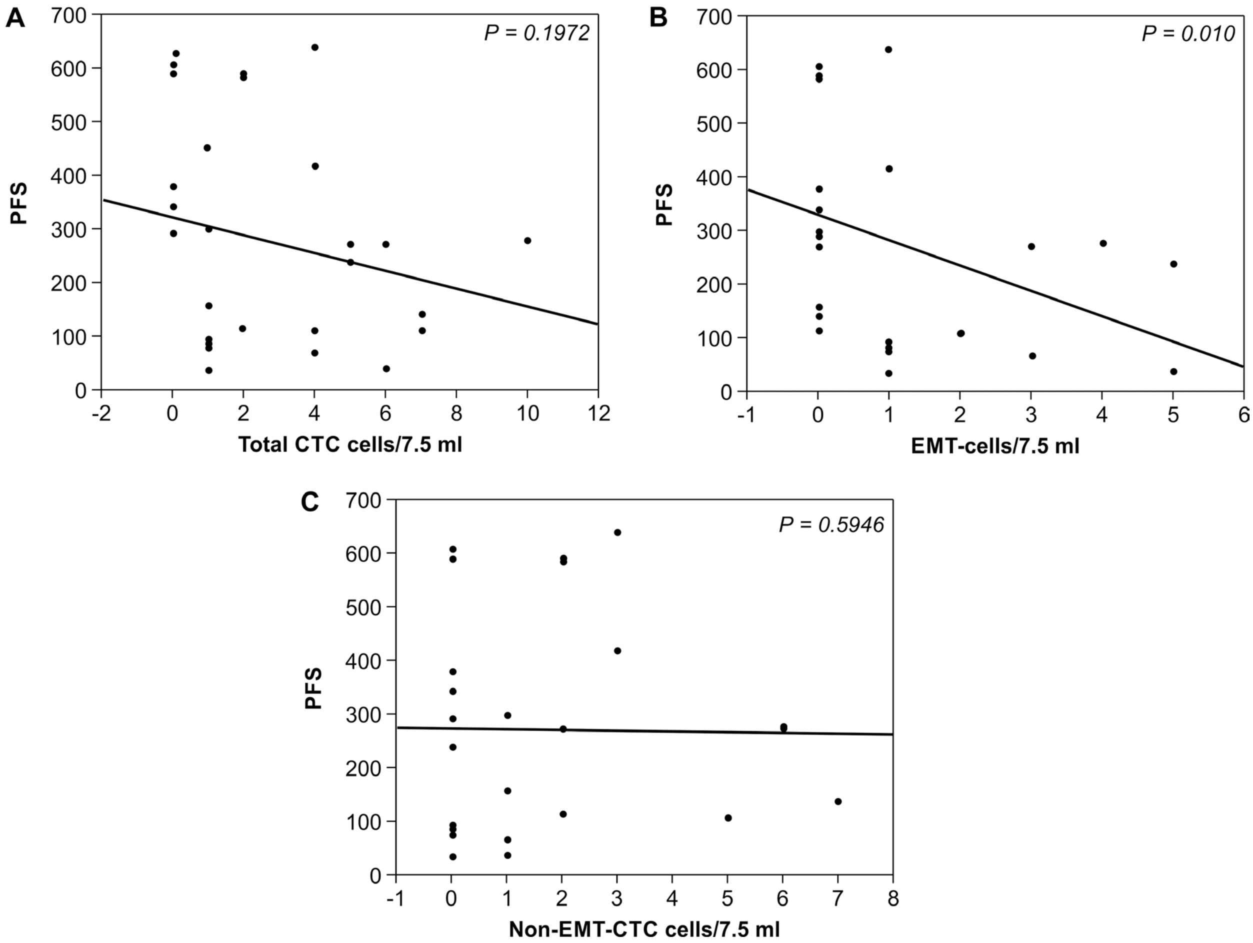

Supplementary Figure 2: Correlation between CTC count in 7.5 mL of peripheral blood from NSCLC patients and PFS (*n* = 30). (**A**) Total CTCs, (**B**) EMT-CTCs, (**C**) Non EMT-CTCs.

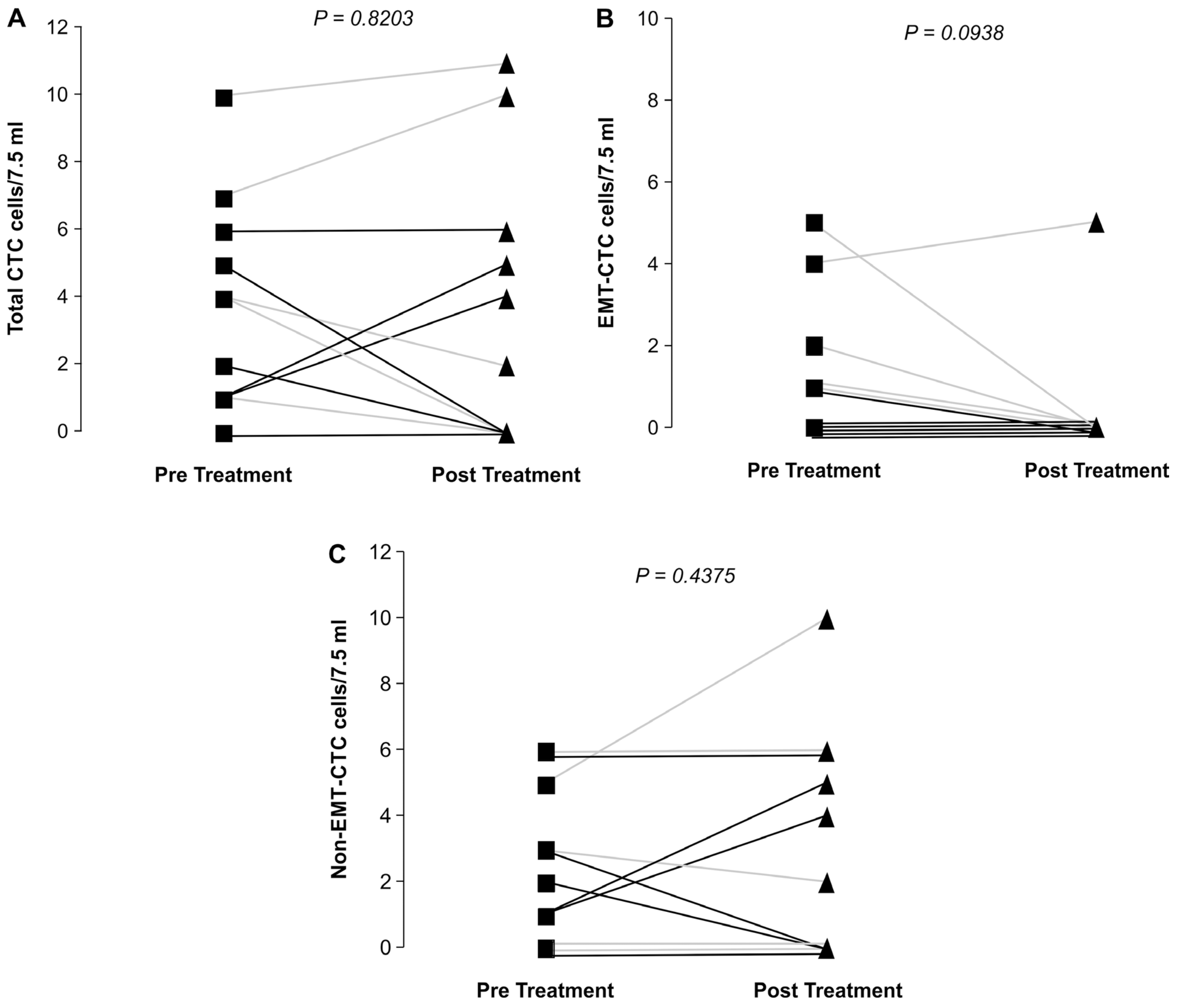

Supplementary Figure 3: CTC change after one cycle of chemotherapy CTC numbers before and after 1 cycle of standard chemotherapy are presented. Serial samples were available for all patients included in this analysis ($n = 11$). (**A**) Total CTCs, (**B**) EMT-CTCs, (**C**) Non EMT-CTCs. Responders (CR or PR) are indicated with black lines and non-responders (SD or PD) are indicated with gray lines.

Supplementary Table 1: Sensitivity of CTC detection according to pathological classification

Pathological classification		Total CTC		EMT-CTC		Non-EMT-CTC	
		CTC positive pts (%)	*p* values	CTC positive pts (%)	*p* values	CTC positive pts (%)	*p* values
Vessel invasion	(+)	15/20 (75.0%)		12/20 (60.0%)		6/20 (30.0%)	
	(−)	40/63 (63.5%)	0.343	24/63(38.1%)	**0.085**	22/63(34.9%)	0.6851
Lymphovascular	(+)	11/18 (67.7%)		7/18 (38.9%)		6/18(33.3%)	
invasion	(−)	44/63 (63.5%)	0.6012	29/65(44.6%)	0.6644	22/65(33.9%)	0.9675
Histlogical Grade							
	Grade1	18/31 (58.1%)		12/31 (38.7%)		9/31 (29.0%)	
	Grade2 ~	42/57 (73.7%)	0.1329	28/57 (49.1%)	0.3487	24/57 (42.1%)	0.2263
Histological Predominancy							
	Non-solid	41/63 (65.1%)		25/63 (39.7%)		23/63 (36.5%)	
	Solid	4/6 (66.7%)	0.4001	2/6 (33.3%)	0.6941	2/6 (33.3%)	0.7454
	Lepi	12/20 (60.0%)		10/20 (50.0%)		6/20 (30.0%)	
	Non-Lepi	33/50 (66.0%)	0.636	17/50 (34.0%)	0.2141	19/50 (38.0%)	0.528
